# Spontaneous Discharge of a Large Vesical Calculus from a Male

**Published:** 1880-06

**Authors:** Charles G. Stockton

**Affiliations:** Buffalo, N. Y.


### SPONTANEOUS DISCHARGE OF A LARGE VESICAL CALCULUS FROM A MALE.

REPORTED BY CHARLES G. STOCKTON, M. D., BUFFALO, N. Y.

Two years since a man entered the Buffalo General Hospital for the purpose of having removed a number of calculi which were confined within his urethra, between its membranous portion and the meatus.

The case was placed in charge of Dr. C. C. F. Gay, at whose suggestion this report is made.

The meatus having been enlarged by a slight incision made near the frænum, permitted the escape of eight or ten uric acid calculi, nearly uniform in size, and about four millimeters in length by two or three in width. No dressing was thought necessary. Much of the history of this case seems very unusual, and is therefore given below.

An American, aged forty-five; when in his twenty-first year, experienced symptoms of vesical calculus, and consulted Dr. W., of Whitehall, N. Y., who examined the patient and informed him that he had a stone in the bladder, and that he could be relieved only by the use of the knife. No operation was made; but after six months of inconvenience the symptoms occasioned by the stone gradually subsided, and finally all disappeared.

Four years later, in 1859, the patient entered the army, serving on the plains of Nebraska, and was most of the time in the saddle. He also contracted syphilis, but enjoyed immunity from his old trouble five years more, when it re-appeared; and he again suffered the ordinary subjective signs of stone in the bladder.

In the year 1865 he returned to this State, and underwent the operation for lithotripsy, in Troy, which resulted in his great relief. In the year 1872 a tumor appeared in the hypogastrium immediately over the pubes, in the median line. It opened externally and discharged, with other matter, a *calculus fully three-fourths of an inch in diameter;* then healing, without any signs of urinary extravasation, left a deep ugly cicatrix. From this time until their removal by Dr. Gay, the patient was troubled with the urethral calculi; since which he pronounces himself well.

The most remarkable, and to me, wonderful part of the above history, is the spontaneous discharge of a large calculus from the bladder of a man; which procedure is surely a novelty in the annals of natural repair. One would feel some incredulity regarding his statement but for these facts. The cicatrix was of very unusual depth. It was removed from the tract of lymphatics, and could not have resulted from a suppurating gland.

The patient was very simple and direct in his statements, and impressed one as a man telling the truth.

He extracted the calculus from the opening with his fingers, and it was for some time preserved. Other facts that confirm the story were obtained by Dr. W. L. Dickinson, one of the hospital internes.

How can we explain such a phenomenal process? May not the stone have been encysted in the anterior wall of the bladder as the first step; then acting as a foreign body, excited inflammation and suppuration till it finally effected its escape, without extravasation of urine? If this stone were not encysted the case might be an argument in favor of the operation for lithotomy. In looking up the literature of supra-pubic openings into the bladder I have found some very interesting statements and statistics pertaining to this operation given by Dr. Dulles, of Philadelphia, in the *American Journal of Medical Sciences*, for April, 1878.

